# Epidemiology of *Chlamydia pneumoniae* infection in children with acute respiratory tract infections, Chengdu, 2022–2023

**DOI:** 10.3389/fpubh.2026.1729558

**Published:** 2026-03-11

**Authors:** Yifei Duan, Yu Wu, Yu Gou, Xiao-Qin Liu, Zheng-Xiang Gao

**Affiliations:** 1 Department of Laboratory Medicine, West China Second University Hospital, Sichuan University. Chengdu, Sichuan, China; 2 Key Laboratory of Birth Defects and Related Diseases of Women and Children, Sichuan University, Ministry of Education, Chengdu, Sichuan, China

**Keywords:** acute respiratory tract infections, children, China, *chlamydia pneumoniae*, epidemiology

## Abstract

**Background:**

*Chlamydia pneumoniae* is a common pathogen involved in acute respiratory tract infections (ARTIs) in children.

**Methods:**

Children with ARTIs attending the West China Second Hospital of Sichuan University from January 2022 to December 2023 were selected. IgM antibodies against Chlamydia pneumoniae (*C. pneumoniae*) were detected using a chemiluminescence immunoassay. Demographic and temporal differences in IgM positivity were compared between 2022 and 2023.

**Results:**

Over the two-year period, 20,689 children were included. In 2022, under stringent COVID-19 non-pharmaceutical interventions (NPIs), 453 positive and 10,092 negative cases, with IgM seropositivity rate of 4.3%. In 2023, following the nationwide relaxation of these NPIs, 602 positive cases, and 9,542 negative cases for IgM seropositivity rate of 5.9%, which was significantly higher than that in 2022 (*P* < 0.001). The IgM seropositivity rate of *C. pneumoniae* infection in children aged 3 to 14 years was significantly higher in 2023 than in 2022 (*P* < 0.05). The IgM seropositivity rate from January to May 2023 was significantly higher than that during the same period of 2022 (*P* < 0.001), while the IgM seropositivity rate from July to October 2023 was significantly lower than that during the same period in 2022 (*P* < 0.05). The IgM seropositivity rate in females aged 3 to 6 years was significantly higher than that of males in the same age group (*P* < 0.05). With the exception of 0- to 1-year-olds, the IgM seropositivity rates of females were significantly higher than those of males in the same age groups in 2023 (*P* < 0.05). Moreover, the IgM seropositivity rate of females was significantly higher than that of males in March, June, and September in 2022 (*P* < 0.05), and the IgM seropositivity rate of females was significantly higher than that of males in the same period in January, May, October, and November in 2023 (*P* < 0.05).

**Conclusion:**

The results revealed that the epidemic trend and population susceptible to *C. pneumoniae* changed from 2022 to 2023, providing valuable insights into the prevention, diagnosis and management of *C. pneumoniae* infection in this region.

## 1 Introduction

Acute respiratory tract infections (ARTIs) are the most prevalent illness in both children and adults and affects all age groups and sexes, during all seasons and in all regions. It is a major cause of morbidity and mortality from infectious diseases both in China and worldwide (1). Children, owing to their immature immune systems and inadequate personal protection, are relatively susceptible to infection by respiratory pathogens, making them particularly vulnerable to viral and bacterial infections. This poses a significant challenge to public health (2, 3). ARTIs can be caused by a variety of pathogens, including viruses, bacteria, *Mycoplasma* spp., *Chlamydia* spp., fungi, and parasites. Viral pathogens include influenza virus, respiratory syncytial virus, rhinovirus, and others (4, 5). Chlamydia pneumoniae (*C. pneumoniae*) is a common pathogen involved in respiratory infections in children. On average, approximately 10% of community-acquired pneumonia is caused by *C. pneumoniae* infection. Before the age of 20, almost all people are infected with *C. pneumoniae* (6). As a result, monitoring *C. pneumoniae* transmission has gained increasing attention. Surveillance helps to understand the distribution of *C. pneumoniae* in the population, track its epidemic trends, provide early warnings for potential outbreaks of *C. pneumoniae*-induced respiratory infections, and offer a scientific basis for clinical diagnosis, a reduction in antibiotic misuse, effective treatment, and prevention.

Coronavirus disease 2019 (COVID-19) is caused by severe acute respiratory syndrome coronavirus 2 (SARS-CoV-2), which was first identified in Wuhan, China, in December 2019 and quickly spread, ultimately becoming a worldwide epidemic. The World Health Organization declared COVID-19 a Public Health Emergency of International Concern on January 30, 2020. To control the spread of the virus, a series of non-pharmaceutical interventions (NPIs) were implemented, including social gathering bans, mask-wearing, home quarantining, social distancing, and hand hygiene (7). These measures have been shown to be effective at controlling the spread of SARS-CoV-2 (8). Moreover, these measures influenced the epidemiological trends and transmission patterns of other airborne or fecal-orally transmitted infectious diseases, including the common cold, gastroenteritis, bronchiolitis, and acute otitis media (9). Following the comprehensive lifting of COVID-19 prevention and control measures at the end of 2022, related public health interventions were also canceled.

While the “rebound” epidemiology of viruses (e.g. RSV, influenza) post-NPIs has been documented, data on atypical bacteria, particularly *C. pneumoniae* in children remain sparse and inconsistent. Both *C. pneumoniae* and SARS-CoV-2 are respiratory pathogens that can be transmitted via respiratory droplets and share several common risk factors (10). *C. pneumoniae* is an obligate intracellular bacterium, whose only known natural host is humans. Compared with that of *Mycoplasma pneumoniae*, the biological activity of *C. pneumoniae* is poor; *C. pneumoniae* often presents with non-specific clinical symptoms, poses greater challenges in terms of detection, and can easily delay the diagnosis and treatment of patients (11, 12). As a result, a significant portion of *C. pneumoniae*-infected patients are missed or misdiagnosed (13).

Our research group previously analyzed and published the epidemiological characteristics of respiratory viruses in children with ARTIs before and during the COVID-19 pandemic (14, 15). Currently, research on the epidemiological features of *C. pneumoniae* in children after the relaxation of COVID-19 containment measures is limited. It remains unclear whether the incidence, age distribution, and seasonal patterns of *C. pneumoniae* infections in children changed during the post-pandemic period. Therefore, we conducted a retrospective analysis of *C. pneumoniae* test results from pediatric ARTI patients over a 2-year period, encompassing 1 year under stringent NPIs (2022) and 1 year after their nationwide discontinuation (2023). By analyzing populations with higher IgM seropositivity rates, seasonal trends, and affected age groups, this study aims to provide a reference for the prevention of *C. pneumoniae* across different seasons and in different age groups and provide a scientific basis for the clinical diagnosis, effective treatment, and control of *C. pneumoniae*-associated ARTIs in children.

## 2 Materials and methods

### 2.1 Study population

**Figure 1:**
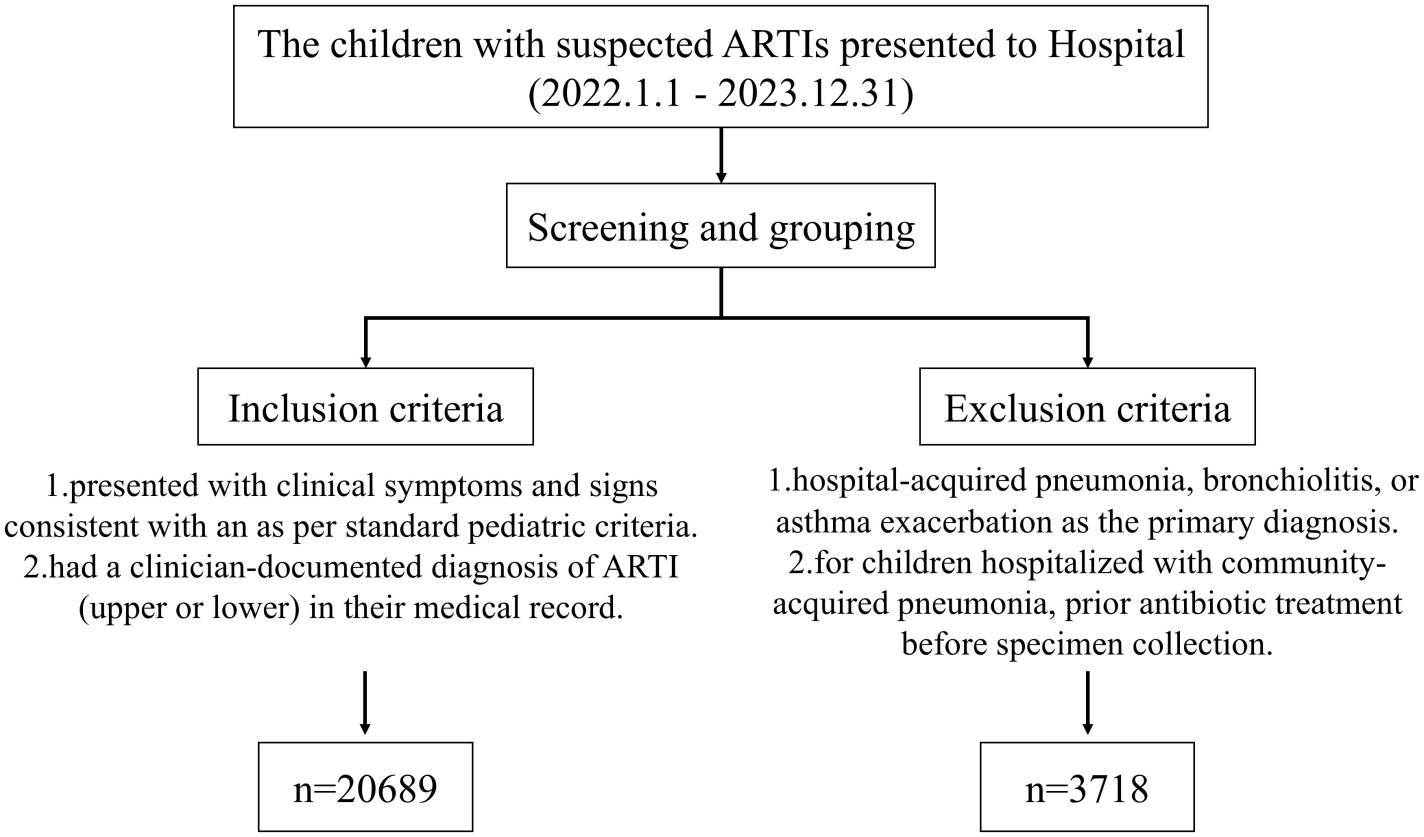
Flow diagram of patient screening and inclusion.

We retrospectively analyzed data from children with suspected ARTIs who presented to West China Second University Hospital, Sichuan University, from January 1, 2022, to December 31, 2023. The 2022 represents the period during which stringent COVID-19 NPIs were fully implemented in China. The 2023 represents the period after the major nationwide relaxation of these NPIs in December 2022. The inclusion criteria were as follows: (1) presented with clinical symptoms and signs consistent with an ARTI (e.g., cough, fever, rhinorrhea, sore throat) as per standard pediatric criteria (16); and (2) had a clinician-documented diagnosis of ARTI (upper or lower) in their medical record. Exclusion criteria included: (1) hospital-acquired pneumonia, bronchiolitis, or asthma exacerbation as the primary diagnosis; and (2) for children hospitalized with community-acquired pneumonia, prior antibiotic treatment before specimen collection (17) (Figure 1).

### 2.2 Sample evaluation

Serum was collected from patients sent to the hospital laboratory for testing. After receiving the serum, laboratory technicians immediately processed and tested the samples. *Chlamydia pneumoniae* IgM antibodies were detected by a *C. pneumoniae* IgM CLIA microparticle assay (Autobio Diagnostics Co., Ltd., China). According to the manufacturer's instructions, an IgM value > 1.0 S/CO was considered positive.

### 2.3 Data collection

Laboratory test results and patient demographic data (name, sex, age, clinical diagnosis, sample collection time) were extracted from the Hospital's Laboratory Information System (LIS). The study protocol was approved by the Ethics Committee of West China Second University Hospital, Sichuan University. The approval number: Medical Research Ethics Approval (290) of 2023.

### 2.4 Statistical analysis

Statistical analysis was performed using SPSS 26.0 software. Categorical variables are expressed as numbers and percentages (*n*, %). All variables, including age and sex, were tested for a normal distribution. Owing to the unequal population size and data differences, the chi-square test or Fisher's exact test was used to compare differences between groups (months, sex, and age groups), and a *P* value < 0.05 was considered to indicate statistical significance.

## 3 Results

### 3.1 Population characteristics

Table 1 and Supplementary Table S1 summarize the sociodemographic characteristics of the patients from whom samples were taken. A total of 20,689 children with ARTIs were tested for *C. pneumoniae* from 2022 to 2023. In 2022, under stringent COVID-19 NPIs, there were 10,545 cases, and in 2023, following the nationwide relaxation of these NPIs, there were 10,144 cases. With respect to age distribution, children aged 3–6 years represented the largest group tested in both 2022 and 2023, followed by those aged 6–14 years and 1–3 years. Children aged 0–30 days had the fewest tests. In 2022, 4,549 children aged 3–6 years were tested, representing 43.1% of the total, and 9 children aged 0–30 days, representing 0.1%. In 2023, 4,224 children aged 3–6 years were tested (41.6% of the 2023 total) as well as 11 children aged 0–30 days (0.1%). In 2022, there were 5,584 male children (53.0%) and 4,961 female children (47.0%) tested, with a male-to-female ratio of 1.13:1. In 2023, there were 5,098 male children (50.3%) and 5,046 female children (49.7%) tested, with a male-to-female ratio of 1.01:1. The number of children aged 30 days to 1 year and 6 to 14 years tested in 2022 was significantly greater than that in 2023 (*P* < 0.001), whereas the number of children aged 1–3 years and >14 years tested in 2023 was significantly greater than that in 2022 (*P* < 0.001). In 2022, there were 9,645 outpatients (91.5%) and 900 inpatients (8.5%), with an outpatient-to-inpatient ratio of 10.7:1. In 2023, there were 9,593 outpatients (94.6%) and 551 inpatients (5.4%), with an outpatient-to-inpatient ratio of 17.3:1. The number of outpatient tests was significantly greater than the number of inpatient tests across both years. With respect to temporal distribution, in 2022, the highest number of tests occurred over 6 months (January, March–July), accounting for 67.1% of the annual total. September had the fewest tests, with 242 cases (2.3%). In 2023, the highest number of tests occurred over 5 months (April, June–July, and October–November), accounting for 52.7% of the annual total. The fewest tests were performed in February, with 349 cases (3.4%). The number of tests from January to June 2022 was significantly greater than that in 2023 (*P* < 0.05), while the number of tests from August to December 2023 was significantly greater than that in 2022 (*P* < 0.001).

**Table 1:** The sociodemographic variables of the study subjects.

**Age**	**2022 (*n* = 10,545)**	**2023 (*n* = 10,144)**	**χ^2^ value**	***P* value**
0–30 d	9 (0.1)	11 (0.1)	0.29	0.593
30 d−1 y	773 (7.3)	590 (5.8)	19.26	< 0.001
1–3 y	1,968 (18.7)	2,179 (21.5)	25.62	< 0.001
3–6 y	4,549 (43.1)	4,224 (41.6)	4.75	0.029
6–14 y	3,108 (29.5)	2,293 (22.6)	126.47	< 0.001
>14 y	138 (1.3)	847 (8.3)	565.31	< 0.001
**Gender**				
Male	5,584 (53.0)	5,098 (50.3)	15.07	< 0.001
Female	4,961 (47.0)	5,046 (49.7)		
**Patient**				
Outpatient	9,645 (91.5)	9,593 (94.6)	96.34	< 0.001
Inpatient	900 (8.5)	551 (5.4)		

The percentage was calculated as: group samples/annual total samples × 100%.

### 3.2 The characteristics of *C. pneumoniae*-positive children

**Figure 2:**
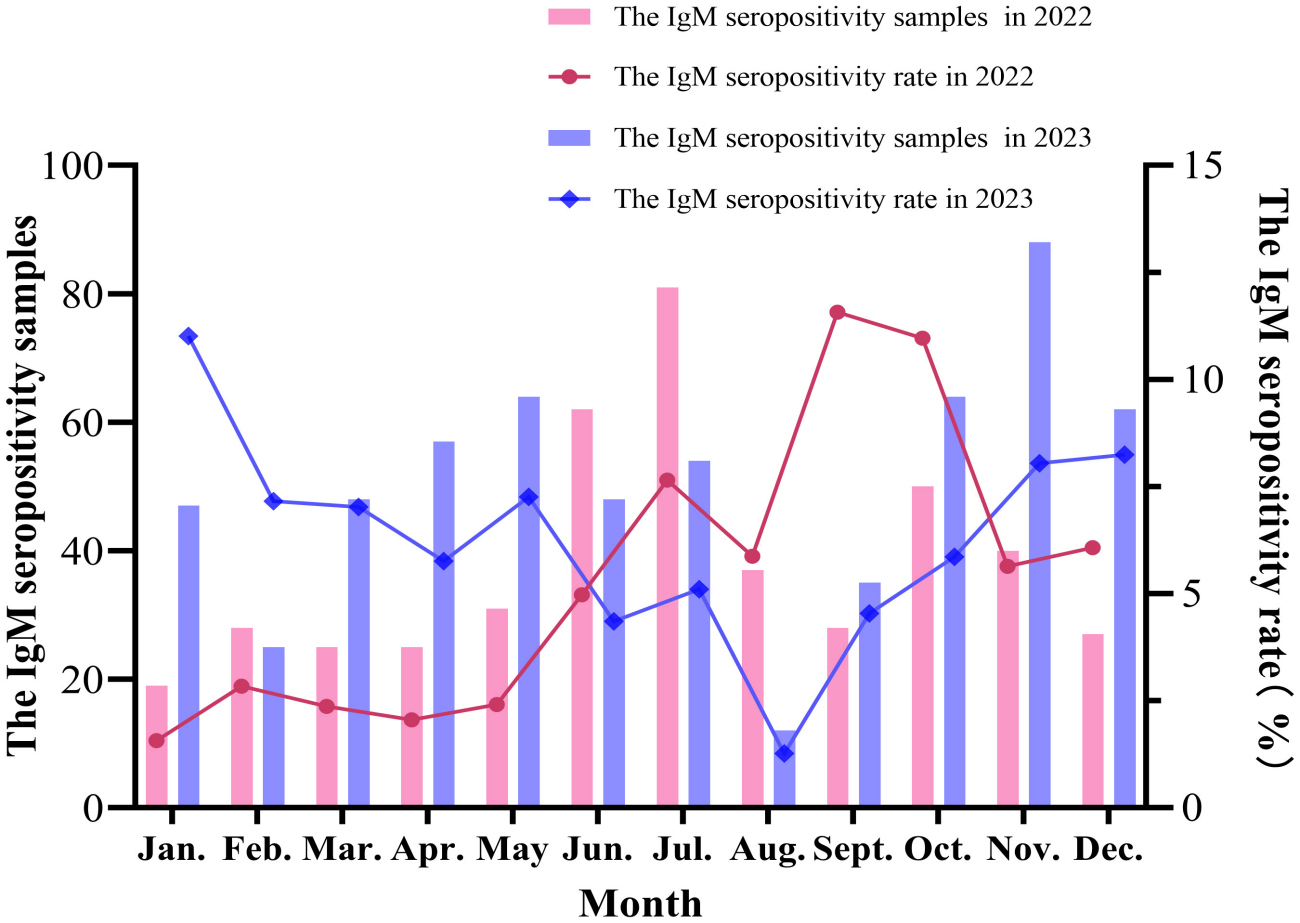
The monthly IgM seropositivity of C. pneumoniae from January 1, 2022 to December 31, 2023.

In 2022, there were 453 positive cases and 10,092 negative cases of *C. pneumoniae* infection, with a positive-to-negative ratio of 0.04:1. In 2023, there were 602 positive cases and 9,542 negative cases, with a positive-to-negative ratio of 0.06:1. Among these, there were 205 positive males and 248 positive females in 2022, with a male-to-female positive ratio of 0.83:1, and there were 231 positive males and 371 positive females in 2023, with a male-to-female positive ratio of 0.62:1. There were more positive females than males across both years. In 2022, there were 396 positive outpatients and 57 positive inpatients, with an outpatient-to-inpatient positive ratio of 6.95:1. In 2023, there were 572 positive outpatients and 30 positive inpatients, with an outpatient-to-inpatient positive ratio of 19.07:1. The number of positive outpatients was significantly higher than that of positive inpatients across both years. With respect to the age distribution of positive cases, in 2022, children aged 0–30 days had the highest IgM seropositivity rate (11.1%), but the total number tested in this group was only 9. The next highest rate was in the >14-year-old group (8.0%), while the 3–6-year-old group had the lowest IgM seropositivity rate (3.6%). In 2023, the >14-year-old group had the highest IgM seropositivity rate (9.6%), and the 0–30-day-old group had the lowest (0.0%). The IgM seropositivity rate for *C. pneumoniae* in children aged 3–14 years in 2023 was significantly higher than that in 2022, and the difference was statistically significant (*P* < 0.05). The hospitalization rate of IgM seropositivity patients was 12.58% in 2022 compared to 4.98% in 2023 (*P* < 0.001). This indicates that the positive cases enrolled in 2022 represented a clinically more severe patient population than those in 2023 (Table 2). With respect to the temporal distribution, the IgM seropositivity rate was highest in September (11.6%) and lowest in January (1.6%) in 2022, and the IgM seropositivity rate was highest in January (11.0%) and lowest in August (1.3%) in 2023. Except for June, November, and December, the differences in the monthly IgM seropositivity rate s were statistically significant across the 2 years. Specifically, the IgM seropositivity rates from January to May in 2023 were significantly higher than those in the same period in 2022 (*P* < 0.001), while the IgM seropositivity rates from July to October in 2023 were significantly lower than those in the same period in 2022 (*P* < 0.05) (Figure 2 and Supplementary Table S2).

**Table 2:** The characteristics of children with IgM seropositivity of *C. pneumoniae*.

**Age**	**Positive (*n*)**	**%, (95% CI)**	**Positive (*n*)**	**%, (95% CI)**	**χ^2^**	***P***
	**2022 (*****n*** = **453)**		**2023 (*****n*** = **602)**			
0–30 d	1	11.1 (−9.4–31.6)	0	0	1.29	0.450
30 d−1 y	30	3.9 (2.5–5.2)	18	3.1 (1.7–4.4)	0.68	0.410
1–3 y	72	3.7 (2.8–4.5)	81	3.7 (2.9–4.5)	0.01	0.920
3–6 y	165	3.6 (3.1–4.2)	206	4.9 (4.2–5.5)	8.45	0.004
6–14 y	174	5.6 (4.8–6.4)	216	9.4 (8.2–10.6)	28.76	< 0.001
>14 y	11	8 (3.5–12.5)	81	9.6 (7.6–11.5)	0.36	0.551
**Gender**						
Male	205	1.9 (1.7–2.2)	231	2.3 (2–2.6)	5.03	0.025
Female	248	2.4 (2.1–2.6)	371	3.7 (3.3–4)	23.88	< 0.001
**Patient**						
Outpatient	396	3.8 (3.4–4.1)	572	5.6 (5.2–6.1)	34.71	< 0.001
Inpatient	57	0.5 (0.4–0.7)	30	0.3 (0.2–0.4)	0.48	0.489

*n*, the total positive samples.

### 3.3 Differences in age and temporal distribution by sex among *C. pneumoniae*-positive children

In 2022, the IgM seropositivity rate in females aged 3–6 years was significantly higher than that in males of the same age group (*P* < 0.05), with no significant differences across other age groups (Table 3). In 2023, except for the 0–1 year age group, the IgM seropositivity rates in females were significantly higher than those in males of the same age groups (*P* < 0.05) (Table 4). With respect to the monthly temporal distribution by sex, in 2022, the IgM seropositivity rates in females were significantly higher than those in males of the same age groups during March, June, and September *(P* < 0.05), with no significant difference in the remaining months (Table 3). In 2023, the IgM seropositivity rates in female children were significantly higher than those in males during January, May, October, and November (*P* < 0.05), with no significant difference in the remaining months (Table 4).

**Table 3:** Differences in age and temporal distribution by sex among *C. pneumoniae*- IgM seropositivity children in 2022.

**Age**	**Positive (*n*)**	**%, (95% CI)**	**Positive (*n*)**	**%, (95% CI)**	**χ^2^**	***P***
	**Male (*****n*** = **205)**		**Female (*****n*** = **248)**			
0–30 d	1 (4)	11.1 (−9.4–31.6)	0 (5)	0	1.41	0.444
30 d−1 y	19 (459)	2.5 (1.4–3.5)	11 (314)	1.4 (0.6–2.3)	0.20	0.653
1–3 y	34 (1,096)	1.7 (1.2–2.3)	38 (872)	1.9 (1.3–2.5)	2.17	0.141
3–6 y	67 (2,318)	1.5 (1.1–1.8)	98 (2,231)	2.2 (1.7–2.6)	7.34	0.007
6–14 y	79 (1,633)	2.5 (2–3.1)	95 (1,475)	3.1 (2.5–3.7)	3.77	0.052
>14y	5 (74)	3.6 (0.5–6.7)	6 (642)	4.3 (0.9–7.8)	0.32	0.571
**Month**					
Jan.	7 (624)	0.6 (0.2–1)	12(586)	1.0 (0.4–1.6)	1.68	0.195
Feb.	18 (547)	1.8 (1–2.7)	10(440)	1.0 (0.4–1.6)	0.92	0.338
Mar.	8 (562)	0.8 (0.2–1.3)	17(494)	1.6 (0.9–2.4)	4.63	0.031
Apr.	13 (650)	1.1 (0.5–1.6)	12 (596)	1.0 (0.4–1.5)	0.02	0.893
May	12 (684)	0.9 (0.4–1.5)	19 (600)	1.5 (0.8–2.1)	2.71	0.100
Jun.	25 (659)	2.0 (1.2–2.8)	37 (589)	3.0 (2–3.9)	4.08	0.043
Jul.	38 (579)	3.6 (2.5–4.7)	43 (480)	4.1 (2.9–5.2)	2.13	0.144
Aug.	17 (314)	2.7 (1.4–4)	20 (316)	3.2 (1.8–4.5)	0.24	0.625
Sept.	10 (137)	4.1 (1.6–6.6)	18 (105)	7.4 (4.1–10.7)	5.63	0.018
Oct.	22 (248)	4.8 (2.9–6.8)	28 (208)	6.1 (3.9–8.3)	2.44	0.118
Nov.	17 (344)	2.4 (1.3–3.5)	23 (365)	3.2 (1.9–4.5)	0.62	0.433
Dec.	18 (236)	4.0 (2.2–5.9)	9 (209)	2.0 (0.7–3.3)	2.15	0.143

*n*, The total samples of this group. %: positive samples/total samples of this group × 100%.

**Table 4:** Differences in age and temporal distribution by sex among *C. pneumoniae*- IgM seropositivity children in 2023.

**Age**	**Positive (*n*)**	**%, (95% CI)**	**Positive (*n*)**	**%, (95% CI)**	**χ^2^**	***P***
	**Male (n**=**232)**		**Female (n**=**372)**			
0–30 d	0 (8)	0	0 (3)	0	/	/
30 d−1 y	13 (364)	2.2 (1–3.4)	5 (226)	0.8 (0.1–1.6)	0.87	0.351
1–3 y	32 (1,240)	1.5 (1–2)	49 (975)	2.2 (1.6–2.9)	8.44	0.004
3–6 y	85 (2,187)	2.0 (1.6–2.4)	121 (2,037)	2.9 (2.4–3.4)	9.59	0.002
6–14 y	95 (1,207)	4.1 (3.3–5)	121 (1,086)	5.3 (4.4–6.2)	7.17	0.007
>14 y	6 (128)	0.7 (0.1–1.3)	75 (719)	8.9 (6.9–10.8)	4.15	0.042
**Month**						
Jan.	15 (223)	3.5 (1.8–5.3)	32 (204)	7.5 (5–10)	8.73	0.003
Feb.	13 (193)	3.7 (1.7–5.7)	12 (156)	3.4 (1.5–5.4)	0.12	0.730
Mar.	22 (354)	3.2 (1.9–4.5)	26 (330)	3.8 (2.4–5.2)	0.73	0.395
Apr.	26 (514)	2.6 (1.6–3.6)	31 (476)	3.1 (2–4.2)	0.96	0.326
May	25 (463)	2.8 (1.7–3.9)	39 (418)	4.4 (3.1–5.8)	5.04	0.025
Jun.	20 (565)	1.8 (1–2.6)	28 (538)	2.5 (1.6–3.5)	1.84	0.176
Jul.	23 (568)	2.2 (1.3–3.1)	31 (490)	2.9 (1.9–3.9)	2.82	0.093
Aug.	6 (519)	0.6 (0.1–1.1)	6 (423)	0.6 (0.1–1.1)	0.13	0.721
Sept.	13 (407)	1.7 (0.8–2.6)	22 (364)	2.9 (1.7–4)	3.60	0.058
Oct.	25 (578)	2.3 (1.4–3.2)	39 (514)	3.6 (2.5–4.7)	5.25	0.022
Nov.	25 (468)	2.3 (1.4–3.2)	63 (627)	5.8 (4.4–7.1)	8.03	0.005
Dec.	19 (246)	2.5 (1.4–3.6)	43 (506)	5.7 (4.1–7.4)	0.13	0.717

*n*, the total samples of this group. %: positive samples/total samples of this group × 100%.

## 4 Discussion

This study was conducted at the West China Second University Hospital of Sichuan University, the largest women and children's specialty hospital in western China, which provides care for a high volume of pediatric patients. Children, as the study population, are particularly susceptible to respiratory tract infections due to their ongoing physical development, including immature respiratory and immune systems, and generally lower compliance with preventive measures such as mask-wearing (18, 19). Respiratory infections are caused by numerous pathogens, primarily viruses and atypical organisms. *C. pneumoniae* often causes respiratory infections and is highly contagious (20). Following the lifting of COVID-19 restrictions, many studies have investigated COVID-19 coinfections with other pathogens, but research on the epidemiological characteristics of *C. pneumoniae* in children remains scarce. The incidence, affected age groups, and seasonal patterns after the lifting of restrictions are still poorly understood. Owing to the complex diversity and overlap of clinical manifestations among respiratory pathogens, laboratory testing is essential (21). Pathogen culture is the gold standard for diagnosing *C. pneumoniae*, but it is rarely used clinically because of its complex procedures, long turnaround times, and vulnerability during specimen collection and to transport conditions. While nucleic acid detection offers high specificity and sensitivity, it requires specialized laboratory equipment and personnel, is costly, is prone to contamination, has limited throughput, and often cannot meet the increasing specimen volume requirements (22, 23). Consequently, simple and rapid detection methods are highly valued by clinicians. The first immunoglobulin to appear in the early stage of infection is IgM; in this study, we primarily detected serum IgM using a CLIA microparticle assay, which is not only simple to perform but also provides high sensitivity and specificity, potentially reducing delays in diagnosis and treatment (24). It is worth noting that there are limitations in using a single serum IgM to detect acute chlamydia pneumoniae infection. While IgM is the first antibody to appear and is useful for indicating recent infection, it can persist for several months and may not be reliably produced upon reinfection. Although the CLIA method used has high specificity, minimal cross-reactivity with other pathogens cannot be entirely ruled out. Therefore, the IgM seropositivity rates reported in this study should be interpreted as markers of recent/reactive infection rather than definitive proof of acute clinical disease at the exact time of testing. Ideally, molecular detection (PCR) or paired serology would provide more definitive evidence of acute infection; however, in our large-scale retrospective clinical setting, these were not routinely feasible due to cost, logistics, and specimen availability constraints.

The majority of the children in this study were outpatients. The number of children seeking medical care in 2023 (the period after lifting strict control measures) slightly decreased compared with that in 2022 (the period under strict control measures), but the IgM seropositivity rate increased substantially. In 2022, 453 children tested positive, accounting for 4.30% of the total tested that year. In 2023, 602 children tested positive, accounting for 5.93%. This increase is likely related to heightened public awareness and protective behaviors during the COVID-19 control period. Measures such as mask-wearing, social distancing, and reduced gatherings decreased children's exposure to pathogens through transmission routes (17, 25). Some scholars have proposed the concept of “immune debt,” suggesting that prolonged implementation of NPIs led to decreased immunity by reducing children's contact with pathogens, potentially resulting in a temporary increase in respiratory infections (26, 27).

In terms of age distribution, the IgM seropositivity rates among children aged 3–14 years were significantly higher in 2023 than in 2022, possibly because of increased time in outdoor activities and higher exposure to pathogens among school-aged children after the lifting of control measures (28). In terms of temporal distribution, the IgM seropositivity rate from January to May 2023 was significantly higher than that in the same period in 2022, whereas the rate from July to October 2023 was significantly lower than that during the same period in 2022. Wang et al. (29) reported that *C. pneumoniae* infection rates peaked in spring and summer, whereas *M. pneumoniae* infections were more prevalent in autumn. Given the gradual lifting of COVID-19 controls in 2023 and the fact that spring and summer are peak seasons for *C. pneumoniae* infection, this likely contributed to the higher IgM seropositivity rate of *C. pneumoniae* from January to May 2023 than during the same period in 2022. Conversely, several studies have indicated that various pathogens exhibited a “staggered peak” trend after the lifting of COVID-19 controls (28). Other studies have suggested that interferons and other host cytokines released during viral infections may inhibit infections by similar viruses (30). After the controls were lifted, the peak periods for *M. pneumoniae* and viral infections were significantly more pronounced in autumn and winter 2023 than in 2022, which may explain the significantly lower positive rates from July to October 2023 than in the same period in 2022.

Previous studies have frequently reported higher infection rates and pathogen detection rates in boys with ARTIs than in girls (14). However, this study revealed that the prevalence of ARTIs and *C. pneumoniae* IgM seropositivity rate was significantly higher among female children than among male children. These findings have also been reported in other studies (31–33). Several factors may contribute to these findings. First, some studies have suggested that female respiratory tract structure may be more sensitive to infection by pathogens (31). This may be related to sex differences in activity patterns and environments. Boys' activities tend to be more outdoor-oriented, whereas girls are more likely to engage in indoor activities; this might contribute to the stronger disease resistance seen in boys (22). second, there may be a correlation between pathogen type and the epidemic season. Some studies have indicated that the incidence of human rhinovirus, influenza, and adenovirus infections is higher in males, whereas the incidence of *M, pneumoniae* infections is higher in females (28, 34). The mechanisms underlying this observed sex difference remain speculative and cannot be determined from our data. Which require further investigation in future studies. It is also important to consider that this finding, from a single-center hospital sample, could be influenced by residual confounding or selection bias, such as potential differences in care-seeking behavior between the families of male and female children.

This study has several limitations. First, it is a retrospective, single-center analysis. Second, the reliance on a single IgM serology test, as discussed above, may not perfectly correlate with acute infection. Third, we focused solely on *C. pneumoniae* and did not investigate co-infections with other pathogens, which are common in ARTIs. Fourth, the exploratory nature led to multiple subgroup comparisons. We did not adjust *P*-values for these multiple comparisons; thus, statistically significant findings from subgroup analyses (particularly monthly variations) should be interpreted as exploratory and require validation. Finally, temporal changes in healthcare-seeking behavior and clinician testing thresholds between the two periods could have influenced the observed IgM seropositivity rates independently of the true infection incidence.

## 5 Conclusion

This study summarizes and analyzes the prevalence of *C. pneumoniae* among children with ARTIs both before and after the lifting of COVID-19 prevention and control measures. Pandemic control measures significantly affected the epidemiological characteristics of *C. pneumoniae* infection. After the controls were lifted, both the number of infections and the IgM seropositivity rate increased significantly compared with those during the period under strict control. Additionally, the IgM seropositivity rate in female children older than 3 years was significantly higher than that in their male counterparts. Clinicians and public health policymakers should monitor changes in the epidemic trends and patterns of *C. pneumoniae* infection and maintain surveillance to prevent potential rebounds and outbreaks of this disease.

## Data Availability

The raw data supporting the conclusions of this article will be made available by the authors, without undue reservation.
