# Supplementary material for: Epidemiology of Chlamydia pneumoniae infection in children with acute respiratory tract infections, Chengdu, 2022–2023
Source: Front Public Health. 2026 Mar 11;14:1729558. doi: 10.3389/fpubh.2026.1729558 (PMC13014552; doi:10.3389/fpubh.2026.1729558)
Supplement: Supplementary file 1 [file Table_1.docx]

Supplementary Table S1 The number of *C. pneumoniae* tests from January 1,2022 to December 31,2023.

| **Month** | | **2022**(n=10545) | **2023**(n=10144) | | **χ^2^ value** | | ***P*** **value** |
| --- | --- | --- | --- | --- | --- | --- | --- |
| Jan. | 1210(11.5) | | 427(4.2) | 374.55 | | ＜0.001 | |
| Feb. | 987(9.4) | | 349(3.4) | 299.92 | | ＜0.001 | |
| Mar. | 1056(10.0) | | 684(6.7) | 71.83 | | ＜0.001 | |
| Apr. | 1219(11.6) | | 990(9.8) | 17.58 | | ＜0.001 | |
| May | 1284(12.2) | | 881(8.7) | 67.27 | | ＜0.001 | |
| Jun. | 1248(11.8) | | 1103(10.9) | 4.75 | | 0.029 | |
| Jul. | 1059(10.0) | | 1058(10.4) | 0.84 | | 0.358 | |
| Aug. | 630(6.0) | | 942(9.3) | 80.77 | | ＜0.001 | |
| Sept. | 242(2.3) | | 771(7.6) | 312.55 | | ＜0.001 | |
| Oct. | 456(4.3) | | 1092(10.8) | 309.83 | | ＜0.001 | |
| Nov. | 709(6.7) | | 1095(10.8) | 107.66 | | ＜0.001 | |
| Dec. | 445(4.2) | | 752(7.4) | 96.72 | | ＜0.001 | |

The percentage was calculated as: monthly samples / annual total samples× 100%.
