# Supplementary material for: Epidemiology of Chlamydia pneumoniae infection in children with acute respiratory tract infections, Chengdu, 2022–2023
Source: Front Public Health. 2026 Mar 11;14:1729558. doi: 10.3389/fpubh.2026.1729558 (PMC13014552; doi:10.3389/fpubh.2026.1729558)
Supplement: Supplementary file 2 [file Table_2.docx]

Supplementary Table S2 The monthly IgM seropositivity of C. pneumoniae from January 1,2022 to December 31,2023.

| **Month** | | **2022**(n=453) | | **2023**(n=602) | **χ^2^ value** | ***P*** **value** |
| --- | --- | --- | --- | --- | --- | --- |
| Jan. | 19/1210(1.6) | | 47/427(11.0) | | 72.64 | ＜0.001 |
| Feb. | 28/987(2.8) | | 25/349(7.2) | | 12.67 | ＜0.001 |
| Mar. | 25/1056(2.4) | | 48/684(7.0) | | 22.33 | ＜0.001 |
| Apr. | 25/1219(2.0) | | 57/990(5.8) | | 21.00 | ＜0.001 |
| May | 31/1284(2.4) | | 64/881(7.3) | | 29.30 | ＜0.001 |
| Jun. | 62/1248(5.0) | | 48/1103(4.4) | | 0.50 | 0.480 |
| Jul. | 81/1059(7.7) | | 54/1058(5.1) | | 5.74 | 0.017 |
| Aug. | 37/630(5.9) | | 12/942(1.3) | | 26.44 | ＜0.001 |
| Sept. | 28/242(11.6) | | 35/771(4.5) | | 15.61 | ＜0.001 |
| Oct. | 50/456(11.0) | | 64/1092(5.7) | | 12.28 | ＜0.001 |
| Nov. | 40/709(5.6) | | 88/1095(8.0) | | 3.74 | 0.053 |
| Dec. | 27/445(6.1) | | 62/752(8.2) | | 1.93 | 0.165 |

The percentage was calculated as: positive samples / total samples of this group× 100%, n：The total positive samples.
